# Isolated Obturator Internus Muscle Strain Injury in a Professional Football Player: A Case Report

**DOI:** 10.7759/cureus.23949

**Published:** 2022-04-08

**Authors:** Alexandros Toliopoulos

**Affiliations:** 1 Sports Medicine, Rehabilitation and Sports Medicine Clinic Evosmos, Thessaloniki, GRC

**Keywords:** strain, muscle, injury, internus, obturator

## Abstract

The present case report concerns an isolated obturator internus muscle strain. The patient, who was a 30-year-old professional, elite-level football player, suffered the injury during the warm-up before a football match. Isolated injuries of the obturator internus are very uncommon and they are extremely rarely reported in the literature. The diagnosis was made clinically, documented by magnetic resonance imaging. The rehabilitation program was conducted conservatively with physical therapy and kinesiotherapy. The patient’s return to full athletic activity took place 2 weeks after the incident.

## Introduction

Muscle strain injuries of the obturator internus muscle are extremely rare. Only a few case reports are mentioned in the literature. The isolated traumatic injury of the obturator internus muscle is even more rarely described. The case reports that have described the injury so far raise the suspicion that there is a correlation with ball kicking sports, such as football and rugby. This case report presents an isolated strain injury in the obturator internus of a professional, elite-level football player.

## Case presentation

A 30-year-old male of Northern European descent, elite-level football player, who plays in the first division of a European League, complained of sudden onset right buttock pain during a warm-up session before an official football match. In particular, he felt acute pain after a strong pass in the lower gluteal area, which was accompanied by mild symptoms of sciatica. The sciatica symptoms have been after a couple of hours resolved. The athlete could not participate in the game and needed to be substituted.

**Figure 1:**
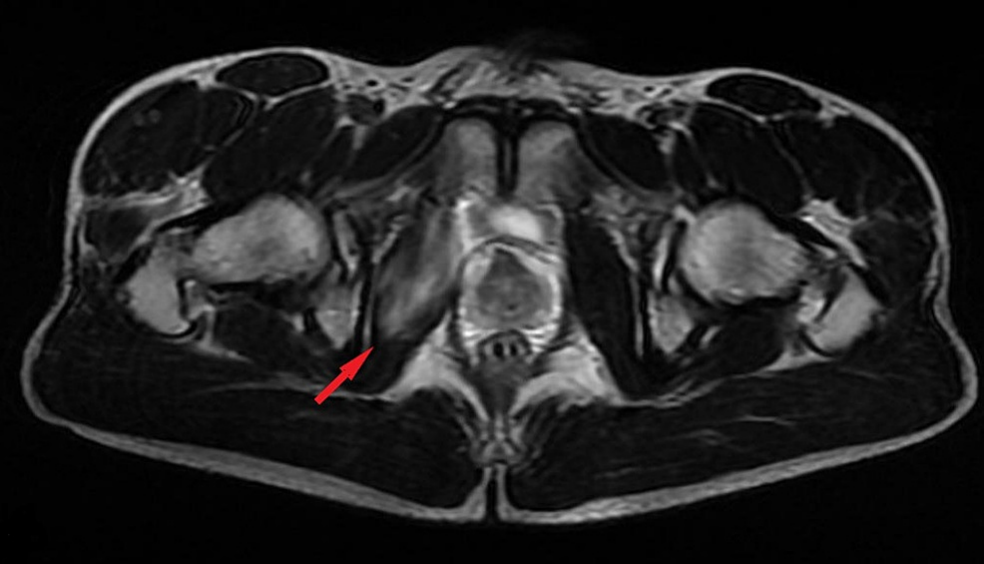
MRI T2 Sequence Axial View FSE Axial view of the right hip at the level of greater trochanter. Hypersignal (Grade II muscle strain) in the obturator internus at the intrapelvic route, with the development of edema and fluid collections around and between its fibers. No other pathology was revealed. The red arrow shows the obturator internus. FSE: Fast Spin Echo

**Figure 2:**
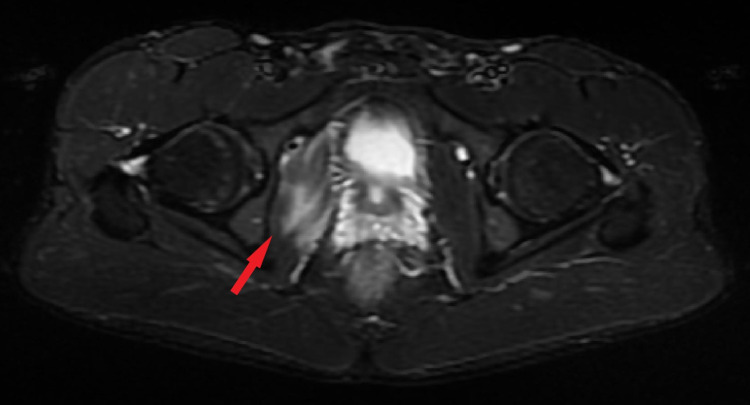
MRI STIR Sequence Axial View Axial view of the right hip at the level of the greater trochanter.  High signal (Grade II muscle strain) in the obturator internus muscle belly at the intrapelvic route. No other pathology was revealed. The red arrow shows the obturator internus. STIR: Short Tau Inversion Recovery Image.

**Figure 3:**
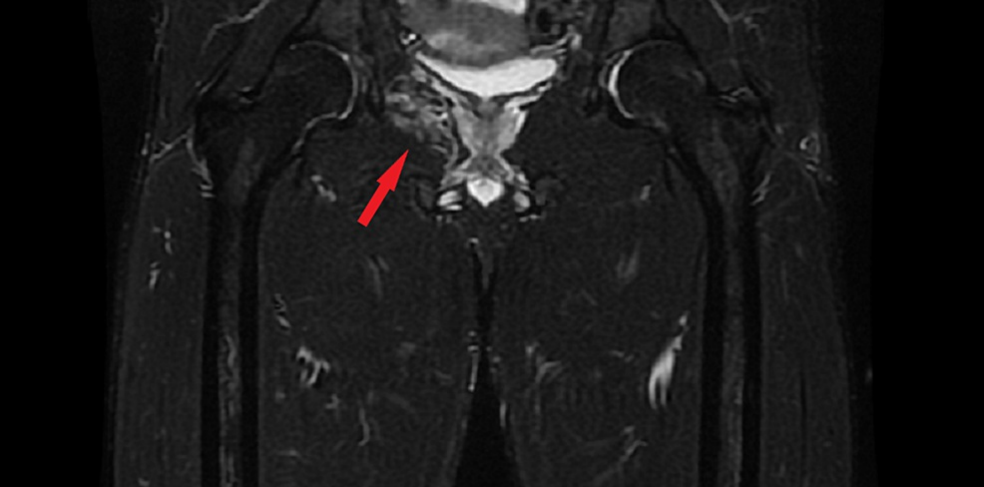
MRI STIR Sequence Coronary View Coronary view of the right hip. Intense signal (Grade II muscle strain) in the obturator internus at the intrapelvic route. The red arrow shows the obturator internus. STIR: Short Tau Inversion Recovery Image

During the examination, there was tenderness on palpation of the lower gluteal area. The range of motion of the right hip was maintained and symmetrical to the contralateral side, but he complained of tenderness during the active external rotation and during the passive stretching of the hip in the internal rotation. The reproduction and exacerbation of the symptoms were pronounced with the hip in adduction. According to his medical history, he suffers from systemic lupus erythematosus and he is on regular medication of chronic anticoagulant treatment with low molecular weight heparin (tinzaparin). He has not had any other hip injuries in his career. The player was referred for an urgent MRI of the hip to delineate any injury of the short external rotators of the hip. On MRI imaging, edema was seen with increased signal intensity in both STIR (short tau inversion recovery) and T2 sequences along almost the entire width of the obturator internus (Figures 1-3). The findings were consistent with second-grade isolated obturator internus strain according to the MRI findings.

Alongside oral anti-inflammatory therapy, an intensive rehabilitation program was followed. Specifically, he abstained from team training and adopted a program that included kinesiotherapy, isometric hip exercises, hydrotherapy, and physical therapy. The patient returned to the individual training program after 6 days and he was able to return to his full competitive activity after 14 days.

## Discussion

The obturator internus is one of the short external rotators of the hip. It is the longest of the external hip rotators with a length of 13.12 ± 0.27 cm [1]. It originates from the inferior margin of the superior pubic ramus and from the pelvic surface of the obturator membrane around the obturator foramen. At its extra pelvic route, the obturator internus passes the lesser ischial foramen alongside the gemellus superior and inferior and they form a common insertion tendon. It inserts onto the medial surface of the greater trochanter of the proximal femur [2]. The obturator internus is innervated by the obturator internus nerve (L5-S2), which branches off the sacral plexus [3]. The main arterial supply is from the inferior gluteal artery [3]. The main action of the obturator internus is the external rotation of the femur when the hip is in a neutral position and at a 90° flexion. Its secondary action is the adduction of the femur when the hip is in flexion [4]. The obturator internus muscle can be considered as a single functional unit together with the superior and inferior gemellus as well as the obturator externus muscle. All together, they are important stabilizers of the pelvis as they stabilize the femoral head to the acetabulum [1].

The reports of pathology of the external rotators in the literature mainly concern chronic syndromes such as the piriformis syndrome. References to traumatic strains of the external rotators are very rare although the obturator internus and the other hip rotators are important pelvic floor muscles [2-10]. This is probably due to the underdiagnosis of such injuries. Most of the cases of obturator internus strain in the literature refer to high-level ball-kicking sports athletes, such as football or rugby players. The possible mechanism of injury is the passing movement of the ball with an externally rotated hip. After literature research, there are only 10 reports of obturator internus strains in ball-kicking sports [2, 3, 5, 6, 7, 8, 9]. One report concerns a young ski athlete [10] and one report concerns an 11-year-old child during a sprint at school [4]. Only a few of these cases report an isolated obturator internus strain.

It is unknown whether the injury is related to the athlete's main disease or his chronic anticoagulant use. While an etiological correlation can be possible, none of the reported cases referred to an athlete under heparin therapy. Clinical signs are pain in the passive internal rotation and in the active external rotation of the hip [7]. Differential diagnosis includes a proximal hamstring injury or an acute onset of chronic piriformis syndrome. MRI is the diagnostic imaging method of choice. MRI diagnosis is important to accurately diagnose the lesion and to rule out other tissue injuries. The athlete of this case report was able to return to full competitive action in 14 days. In all other reported cases in the literature, the disease seemed to be benign with a return-timing to normal activity of 2-6 weeks [7, 8]. The usually reported rehabilitation plan included relative rest and physical means.

## Conclusions

Isolated muscle strain injuries of the obturator internus muscle are extremely rare and it appears that the mechanism of injury is associated usually with ball-kicking sports. Diagnosis of traumatic obturator internus muscle injury can be challenging. After clinical suspicion, an MRI scan is the diagnostic method of choice. The prognosis is usually very good with a quick return to sport after rehabilitation.

## References

[1] Parvaresh KC, Chang C, Patel A, Lieber RL, Ball ST, Ward SR (2019). Architecture of the short external rotator muscles of the hip. BMC Musculoskelet Disord.

[2] Byrne C, Alkhayat A, O'Neill P, Eustace S, Kavanagh E (2017). Obturator internus muscle strains. Radiol Case Rep.

[3] Bisciotti GN, Corsini A, Cena E, Bisciotti AN, Bisciotti AL, Belli A, Volpi P (2019). Acute groin pain syndrome due to internal obturator muscle injury in a professional football player. Joints.

[4] Reintgen C, Bruner M, Smith MS, Moser M (2021). Traumatic obturator internus and quadratus femoris injury in a pediatric patient: a case report. Sports Health.

[5] Wong-On M, Turmo-Garuz A, Arriaza R (2018). Injuries of the obturator muscles in professional soccer players. Knee Surg Sports Traumatol Arthrosc.

[6] Velleman MD, Jansen Van Rensburg A, Janse Van Rensburg DC, Strauss O (2015). Acute obturator internus muscle strain in a rugby player: a case report. J Sports Med Phys Fitness.

[7] Kelm J, Ludwig O, Schneider G, Hopp S (2016). [Injury of the obturator internus muscle--a rare differential diagnosis in a soccer player]. Sportverletz Sportschaden.

[8] Khodaee M, Jones D, Spittler J (2015). Obturator internus and obturator externus strain in a high school quarterback. Asian J Sports Med.

[9] Busfield BT, Romero DM (2009). Obturator internus strain in the hip of an adolescent athlete. Am J Orthop (Belle Mead NJ).

[10] Le HM, Jackson SS (2020). Obturator internus muscle strain in an adolescent skier. J Sports Med Phys Fitness.

